# Outcome of noncritical COVID-19 patients with early hospitalization and early antiviral treatment outside the ICU

**DOI:** 10.3906/sag-2006-173

**Published:** 2021-04-30

**Authors:** Nursel ÇALIK BAŞARAN, Oğuz Abdullah UYAROĞLU, Gülçin TELLİ DİZMAN, Lale ÖZIŞIK, Taha Koray ŞAHİN, Zahit TAŞ, Ahmet Çağkan İNKAYA, Sevilay KARAHAN, Şehnaz ALP, Alpaslan ALP, Gökhan METAN, Pınar ZARAKOLU, Gülay SAİN GÜVEN, Şerife Gül ÖZ, Arzu TOPELİ, Ömrüm UZUN, Murat AKOVA, Serhat ÜNAL

**Affiliations:** 1 Department of Internal Medicine, General Internal Medicine Division,Faculty of Medicine, Hacettepe University, Ankara Turkey; 2 Department of Infectious Diseases and Clinical Microbiology, Faculty of Medicine, Hacettepe University, Ankara Turkey; 3 Department of Internal Medicine, Faculty of Medicine, Hacettepe University, Ankara Turkey; 4 Department of Biostatistics, Faculty of Medicine, Hacettepe University, Ankara Turkey; 5 Department of Microbiology, Faculty of Medicine, Hacettepe University, Ankara Turkey; 6 Department of Internal Medicine, Intensive Care Division, Faculty of Medicine, ,Hacettepe University, Ankara Turkey

**Keywords:** COVID-19, noncritical illness, hydroxychloroquine, favipiravir

## Abstract

**Background/aim:**

Despite the fact that the COVID-19 pandemic has been going on for over 5 months, there is yet to be a standard management policy for all patients including those with mild-to-moderate cases. We evaluated the role of early hospitalization in combination with early antiviral therapy with COVID-19 patients in a tertiary care university hospital.

**Materials and methods:**

This was a prospective, observational, single-center study on probable/confirmed COVID-19 patients hospitalized in a tertiary care hospital on COVID-19 wards between March 20 and April 30, 2020. The demographic, laboratory, and clinical data were collected.

**Results:**

We included 174 consecutive probable/confirmed COVID-19 adult patients hospitalized in the Internal Medicine wards of the University Adult Hospital between March 20 and April 30, 2020. The median age was 45.5 (19–92) years and 91 patients (52.3%) were male. One hundred and twenty (69%) were confirmed microbiologically, 41 (23.5%) were radiologically diagnosed, and 13 (7.5%) were clinically suspected (negative microbiological and radiological findings compatible with COVID-19); 35 (20.1%) had mild, 107 (61.5%) moderate disease, and 32 (18.4%) had severe pneumonia. Out of 171 cases, 130 (74.3%) showed pneumonia; 80 were typical, and 50 showed indeterminate infiltration for COVID-19. Patients were admitted within a median of 3 days (0-14 days) after symptoms appear. The median duration of hospitalization was 4 days (0-28 days). In this case series, 13.2% patients were treated with hydroxychloroquine alone, 64.9% with hydroxychloroquine plus azithromycin, and 18.4% with regimens including favipiravir. A total of 15 patients (8.5%) were transferred to the ICU. Four patients died (2.2%).

**Conclusion:**

In our series, 174 patients were admitted to the hospital wards for COVID-19, 69% were confirmed with PCR and/or antibody test. At the time of admission, nearly one fifth of the patients had severe diseases. Of the patients, 95.4% received hydroxychloroquine alone or in combination. The overall case fatality rate was 2.2%.

## 1. Introduction

As of mid-June, 2020 the number of COVID-19 cases exceeded seven and a half million and more than 425 thousand deaths were reported worldwideWorld Health Organization (2020). Coronavirus Disease (COVID-19) Situation Report [online]. Website: https://www.who.int/docs/default-source/coronaviruse/situation-reports/20200513-covid-19-sitrep-114.pdf?sfvrsn=17ebbbe_4 [accessed 06 06 2020].. Currently, Turkey ranks at 12 in the list of countries with the highest number of cases, with a total number of infected patients reaching 170,000, with more than 4000 deaths in the 3 months after the first COVID-19 case was reported on March 11, 2020Republic of Turkey Ministry of Health (2020). Novel coronavirus actual state, 06 June 2020 [online]. Website: https://covid19.saglik.gov.tr/ [accessed 06 June 2020]. . Despite an initial decline of new emerging cases, the pandemic is far from ceasing and new wave(s) of emerging cases are expected in the wake of removing strict lockdown measures. Since no highly effective antivirals and vaccines are available so far, the management strategies of patients to reduce morbidity and mortality are of utmost importance.

In this report, we present the first cohort of COVID-19 cases in a Turkish university hospital in Ankara where early admission to the hospital and a variety of antiviral drugs are provided. To the best of our knowledge, this is the largest and most detailed report about demographics, clinical, and laboratory characteristics and outcomes of patients diagnosed with probable/confirmed COVID-19 admitted to the intensive care unit (ICU) wards in a Turkish university hospital.

## 2. Materials and methods 

This was a prospective, observational, single-center study on probable and confirmed COVID-19 patients hospitalized in a university hospital for adults. Local ethics committee approval was obtained (GO 20/354). We included adult patients (≥18 years old) hospitalized in COVID-19 wards between March 20 and April 30, 2020. Critically ill patients with sepsis and/or acute respiratory distress syndrome requiring ICU care at the time of admission were excluded. Treatment and discharge decision were made by attending physicians according to the current national guidelines prepared by the Scientific Advisory Committee of the Turkish Ministry of Health Republic of Turkey Ministry of Health Directorate General of Public Health (2020).COVID-19 (SARS-CoV-2 Infection) guide (in Turkish) [online]. Website: https://covid19bilgi.saglik.gov.tr/depo/rehberler/COVID-19_Rehberi.pdf [accessed 14 April 2020]..

The patients were classified into confirmed and probable cases. The ‘confirmed case’ was a patient with positive SARS-CoV-2 RT-PCR from nasopharyngeal swab or a positive SARS-CoV-2 antibody test. The ‘probable case’ was further divided into ‘clinically suspected’ and ‘radiologically diagnosed’ categories. A ‘clinically suspected case’ was defined as a patient with sudden onset of fever, cough, or dyspnea, who had acute respiratory symptoms that could not be explained with any other cause, and who tested negative for SARS-CoV-2 RT-PCR plus a negative pulmonary imaging test3. The ‘radiologically diagnosed’ patient was a clinically suspected case who also had chest imaging findings compatible with COVID-19. 

Microbiological confirmation was performed using nasopharyngeal sampling [3]. Viral nucleic acid isolation from the samples was achieved by using Bio-Speedy vNAT viral nucleic acid buffer (Bioeksen R&D Technologies Ltd, Turkey). The COVID-19 real-time (RT) PCR kit (Bioeksen R&D Technologies Ltd, Turkey) used in this study was designed to detect SARS-CoV-2 causing COVID-19. The kit is applied to nucleic acid isolates from nasopharyngeal swab, oropharyngeal swab, nasopharyngeal aspirate, nasopharyngeal aspirate lavage, bronchoalveolar lavage, and sputum samples. The detection is achieved via one-step reverse transcription and RT-PCR targeting SARS-CoV-2-specific RdRp (RNA-dependent RNA polymerase) gene fragment. The analytical sensitivity and accuracy of the kit are given by the company as 99.4% and 99.0% respectively. If the first RT-PCR test was negative, a second PCR was ordered after 24 to 48 h. If the second PCR test was negative, SARS-CoV-2 total antibody test [COVID-19 IgM/IgG Ab Test Cassette (Colloidal Gold) (Hotgen, P.R.China)] was performed following the directions of the supplier3. Once the patient was admitted, chest imaging was performed by X-ray and/or low-dose computerized tomography (CT) of the chest at a radiology unit allocated for COVID-19 suspected cases. CT scans were evaluated and reported by a radiologist as a routine practice; the findings were classified as negative, typical, or indeterminate for COVID-19 according to the American College of Radiology definitions [1].

We further classified patients into three categories based on the severity of the clinical presentation according to World Health Organization (WHO) classification Republic of Turkey Ministry of Health Directorate General of Public Health (2020). COVID-19 (SARS-CoV-2 Infection) guide (in Turkish) [online]. Website: https://covid19bilgi.saglik.gov.tr/depo/rehberler/COVID-19_Rehberi.pdf [accessed 14 April 2020].: Mild disease was defined as uncomplicated upper respiratory tract viral infection with no documented pneumonia and accompanied by nonspecific symptoms such as fever, fatigue, cough (with or without sputum production), anorexia, malaise, muscle pain, sore throat, dyspnea, nasal congestion, or headache. Patients with pneumonia with no signs of severe pneumonia and no need for supplemental oxygen were classified as “moderate disease”. Severe pneumonia was defined as fever or suspected respiratory infection, plus one of the following: respiratory rate >30 breaths/min; severe respiratory distress; or O2 saturation through pulse oximetry (SpO2) ≤ 93% on room airWorld Health Organization (2020). Clinical Management of Severe Acute Respiratory Infection (SARI) When COVID-19 Diseases is Suspected. Interim Guidance, 13 March 2020 [Online]. Website: https://apps.who.int/iris/handle/10665/331446 [accessed 14 April 2020]..

The demographics (age, sex, contact history with COVID-19, travel history), medical information (concurrent medical illnesses, medications), symptoms (fever, cough, sore throat, dyspnea, myalgia, nasal discharge, sputum, fatigue, smell or taste loss, diarrhea), and the date the symptoms started were recorded according to the patients’ declaration. Vital signs (temperature, pulse, respiration rate, pulse oxygen saturation, and blood pressure) were recorded daily. Laboratory tests for complete blood count, alanine aminotransferase (ALT), aspartate aminotransferase (AST), lactate dehydrogenase (LDH), blood urea nitrogen (BUN), creatinine, C-reactive protein (CRP), procalcitonin, D-dimer, ferritin, fibrinogen, troponin I, creatine kinase (CK), creatine kinase myocardial band (CK-MB), and triglyceride levels were recorded. The need for oxygen support (oxygen via nasal cannula or mask) was noted. The Modified Early Warning Score (MEWS) of the patients was recorded on the admission date [2,3]. The patients were followed until discharge, transfer to the ICU or death in the wards. The patients not discharged or deceased on April 30 were followed up until May 10, 2020.

The treatment regimen for each patient was decided upon by the primary physician and the consulting team from the Department of Infectious Diseases. We divided treatment regimens into three groups: hydroxychloroquine (HQ)-only, HQ plus azithromycin (AZ), and favipiravir (FAV)-containing regimens. FAV was used in combination with, or as a sequential therapy to the first line treatment regimens (HQ ± AZ) in cases with noncritical illness, but extensive bilateral pneumonia. Twelve-derivation electrocardiogram was obtained initially from all hospitalized patients, and ECG monitoring was performed every other day for patients receiving HQ and/or AZ.

Adverse reactions under treatments were defined as nausea/vomiting requiring antiemetic medication, QT prolongation of >500 ms, arrhythmia, elevation of transaminases >100 U/L, and stopping of antiviral therapy due to any adverse effects during follow-up in the wards. 

We compared treatment regimens in terms of the time to defervescence (the return of body temperature to normal (<38.0 °C) during the hospital stay) and time to clinical improvement on therapy. The clinical improvement was defined when any of the following was observed: resolution of fever, dyspnea, oxygen need, respiratory failure, or discharge.

## 2. Statistical analysis 

Continuous variables were given as mean ± standard deviation if normally distributed, and median (minimum–maximum) for nonnormally distributed variables. Categorical variables were summarized as counts and percentages. The treatment groups were compared with the Kruskal–Wallis test and pairwise comparisons. The Dunn test was applied for pairwise comparisons. The relationships between categorical variables were determined using the chi square test. P-value <0.05 was accepted as statistical significance.

## 3. Results 

A total of 174 consecutive patients with probable/confirmed COVID-19 hospitalized in the Internal Medicine wards of the Hacettepe University Adult Hospital between March 20 and April 30, 2020 were included. The median age was 45.5 years (19–92 years) with a preponderance of males (91 patients, 52.3%). Overall, 120 (69%) cases were confirmed microbiologically, 41 (23.5%) were radiologically diagnosed, and 13 (7.5%) were clinically suspected.

The most frequent symptoms were fatigue (n: 127, 72.9%), cough (n: 125, 71.8%), and fever (n: 104, 60%) (Table 1). Less than half of the cases (n: 82, 47.1%) had contact history with a COVID-19 patient. Only one had a history of international travel. According to WHO definitions, 35 (20.1%) had mild disease, 107 (61.5%) had moderate, and 32 (18.4%) had severe disease (Table 1). Median time from the first appearance of symptoms to the first hospital admission was 3 (0–14) days. 

**Table 1:** Demographic characteristics of probable/confirmed COVID-19 patients.

Characteristics	Total	Mild disease	Moderate disease	Severe disease	P-value
Number of cases, %	174 (100)	35 (20.1)	107 (61.5)	32 (18.4)	
Age, years, median range ≥65 years, n (%)	45.5 19–92 24 (13.8)	44 (24–82) 6	42 (19–74) 8	56.5 (20–92) 10	0.003
Sex Male, n (%) Female, n (%)	91 (52.3) 83 (47.7)	21 14	48 59	22 10	0.035
Contact with COVID-19, n (%)	82 (47.1)	16	54	12	0.430
Comorbid condition, n (%) Hypertension Diabetes mellitus COPD/Asthma CAD/CHF Malignancy Pregnancy	33 (18.9) 26(14.9) 16 (9.2) 14 (8) 6 (3.4) 1 (0.6)	9 10 3 5 0 0	13 6 6 4 2 0	10 10 7 5 4 1	0.169 <0.001 0.022 0.282 0.017 NA
Smoking, n (%) Alcohol, n (%)	56 (32.2) 24 (13.8)	10 3	33 12	13 9	0.523 0.078
ACEI/ARB use, n (%) Ibuprofen use, n (%)	18 (10.3) 8 (4.6)	6 1	9 6	3 1	0.543 0.543
Symptoms on admission, n (%) Fatigue Cough Fever Myalgia Dyspnea Sore throat Nasal discharge Sputum Headache Diarrhea Loss of taste and/or smell	127 (72.9) 125 (71.8) 104 (60) 99 (56.9) 42 (24.1) 56 (32.2) 27 (15.5) 25 (14.4) 23 (13.2) 10 (5.7) 8 (4.5)	21 22 17 19 6 13 5 4 6 4 0	80 83 66 62 23 34 18 13 13 3 7	25 20 20 18 13 9 4 8 4 3 1	0.257 0.071 0.608 0.994 0.035 0.859 0.653 0.151 0.930 0.166 0.194
Duration of symptoms prior to hospital admission, days Median (min–max)	3 (0-14)	3 (0-13)	3 (0-14)	5 (1-14)	0.060

COPD: chronic obstructive pulmonary diseases; CAD: coronary artery diseases; CHF: congestive heart failure; ACEI: angiotensin converting enzyme inhibitor; ARB: angiotensin receptor blocker.

The vital signs and MEWS score of probable/confirmed COVID-19 patients at the time of admission and during hospitalization period are given in Table 2. 

**Table 2:** Vital signs and MEWS scores of probable/confirmed COVID-19 patients at the time of hospitalization and during the hospitalization period.

	Total casesn: 174 (%)	Mild diseasen: 35	Moderate diseasen: 107	Severe diseasen: 32	P-value
Duration of fever, n (%) < 2 days 2–5 days >5 days	108 (62.1) 52 (29.9 ) 14 (8)	26 8 1	67 33 7	15 11 6	0.121
MEWS at admission, n (%) 0–1 points >2 points	142 (81.6) 32(18.4)	30 5	100 7	12 20	<0.001
The highest respiratory rate n (%) <24 /min 24–30 /min >30 /min	141 (81) 20 (11.5) 13 (7.5)	31 3 1	99 5 3	11 12 9	<0.001
Oxygen support n (%) Not required Nasal oxygen Oxygen with mask	141 (81) 20 (11.5) 13 (7.5)	31 3 1	100 3 4	10 14 8	<0.001

MEWS: Modified early warning score.

All patients underwent RT-PCR testing for COVID-19 and 25 had antibody testing done 3–5 days after the second negative RT-PCR test results. A hundred and thirteen (64.6%) were RT-PCR–positive (109 in the first, an additional four in the second RT-PCR testing) and seven tested positive for IgM/IgG total antibodies. CT scans of the chest were performed in all cases except for three patients. Three-quarters of them (n: 130, 74.3%) revealed pneumonia; 80 were typical of, and 50 indeterminate for COVID-19 infiltration (Table 3). 

**Table 3:** Diagnostic test results of the probable/confirmed COVID patients.

	Total casesn: 174	Mild diseasen: 35	Moderate diseasen: 107	Severe diseasen: 32	P-value
Positive PCR, n (%) Negative PCR, n (%)	113 (64.9) 61 (35.1)	23 12	72 35	18 14	0.514
Positive antibody test, n Negative antibody test, n	7 18	0 2	5 11	2 5	0.010
Chest X-ray, n (%) Normal Abnormal Not performed	62 (35.6) 80 (46) 32 (18.4)	21 7 7	34 52 21	7 21 4	<0.001
Chest CT, n (%) Normal, no infiltration Typical infiltration Indeterminate infiltration CT not performed	41 (23.5) 80 (46.1) 50 (28.7) 3 (1.7)	35 0 0 0	3 62 40 2	3 18 10 1	<0.001
Diagnosis, n(%) Confirmed Probable Radiologically diagnosed Clinically suspected	120 (69) 54 (31) 41 (23.6) 13 (7.4)	28 12 0 12	71 31 31 0	21 11 10 1	<0.001

CT: Computerized tomography.

Three-thirds of the patients (n: 116, 66.7%) had lymphopenia (<1500 / mm3) , and 39 (22.4%) had severe lymphopenia (<800 / mm3) at the time of admission (Table 4). C-reactive protein was >4 mg/dL in 40 (22.9%), and D-dimer was >1.0 mg/L in 36 (20.6%) of the cases at the beginning of hospitalization. In 19 (10.9%) cases serum ferritin level was >500 µg/L. Significant differences were observed among the three categories of disease (mild, moderate and severe) in regards to white blood cell, lymphocyte, neutrophil count, NLR, serum LDH, BUN, CRP, procalcitonin, ferritin, D-dimer, and troponin I levels (Table 4).

**Table 4:** Initial laboratory test results of the probable/confirmed COVID-19 patients at the time of admission.

Laboratory parameters	Total	Mild disease	Moderate disease	Severe disease	P-value
Hgb, g/dL (mean ± SD)	13.8 ± 1.8	13.6 ± 2.04	14.0 ± 1.5	13.5 ± 2.39	0.256
WBC (/mm3), median (min–max)	5600 (1000–20,900)	6350 (1000–15,400)	5250 (1800–16300)	6050 (2100–20,900)	0.001
LYM (/mm3), (mean ± SD)	1301 ± 641	1312 ± 835	1386 ± 560	1014 ± 531	0.015
NEU (/mm3), median (min–max)	3810 (720–18,750)	4130 (720–13,700)	3260 (740–14.200)	4280 (1300–18,750)	0.002
NLR; median (min–max)	3.1 (0.5–61.5)	3.1 (0.9–20.7)	2.5 (0.15–12.1)	4.6 (1.6–61.5)	<0.001
PLT (/mm3), (mean ± SD)	196000 ±74.190	206000 ± 66.217	194910 ± 74.137	188190 ± 84.105	0.247
CRP; median (min–max)	1.21 (0.1–21.2)	0.81 (0.14–16.9)	0.96 (0.10–24.20)	2.52 (0.50–23.10)	<0.001
ESR; median (min–max)	12 (2–102)	10 (2–102)	11 (2–87)	20 (2–63)	0.223
Procalcitonin; median (min–max)	0.04 (0.01–9.36)	0.04 (0.01–1.01)	0.03 (0.01–0.67)	0.06 (0.01–9.36)	<0.001
D-dimer; median (min–max)	0.44 (0.19–19.5)	0.42 (0.19–5.05)	0.360 (0.19–19.52)	1.01 (0.21–10.39)	<0.001
LDH; median (min–max) (U/L)	193 (96–739)	171 (122–739)	191 (96–639)	239 (140–580)	0.046
Fibrinogen; median (min–max)	375 (118–900)	366 (195–658)	370 (118–827)	448 (137–900)	0.136
Ferritin; median (min–max)	87 (5.8–3248)	61.7 (6.7–2018)	74.3 (5.8–1895)	318 (39–3248)	<0.001
CK ; median (min–max)	81 (7–3249)	83 (16–369)	77 (14–3249)	99 (7–1648)	0.612
CK/MB; median (min–max)	0.9 (0.2–33)	0.9 (0.3–4)	0.9 (0.2–33)	1.3(0.2–5.7)	0.355
Troponin I; median (min–max)	3 (0.7–5397)	3.6 (2.3–35.2)	2.7 (0.7–5397)	5.8 (2.3–53.5)	<0.001
ALT, median (min–max) (U/L)	21 (4–651)	20 (4–651)	23.5 (5–181)	20.5 (7–65)	0.477
AST, median (min–max) (U/L)	26 (8–696)	24 (8–696)	26 (12–141)	28 (15–72)	0.247
Cre; median (min–max), mg/dL	0.74 (0.5–5.7)	0.73 (0.46–5.29)	0.70 (0.41–1,96)	0.84 (0.05–5.73)	0.077
BUN; median (min–max), mg/dL	12 (3–121)	12 (6–39)	11.3 (4–28)	14.4 (5–121)	0.001
TG; median (min–max), mg/dL	89 (30–844)	86 (30–844)	85 (33–454)	98 (45–269)	0.637

Hgb: hemoglobin, WBC: white blood cell, LYM: lymphocyte, NEU: neutrophill, NLR: neutrophil/lymphocyte ratio, PLT: platelet, CRP: C-reactive protein, ESR: erythrocyte sedimantation rate, LDH: lactate dehydrogenase, CK: creatine kinase, CK-MB: creatine kinase myoglobin band, ALT: alanine aminotransferase, AST: aspartate aminotransferase, Cre: creatinine, BUN: blood urea nitrogen, TG: triglyceride.

Multiplex RT-PCR tests for viral (n = 148) and bacterial respiratory pathogens (n = 147) were performed from nasopharyngeal swab samples using Allplex Respiratory Panel (Seegene, South Korea). Only 5 samples (3.4%) were positive for another viral pathogen, and 19 samples (12.9%) were positive for at least one bacterial pathogen, in 23 COVID-19 patients (Table 5). Only in one clinically suspected patient nasopharyngeal swab PCR was positive for *H. influenzae.* The patients with a positive bacterial PCR test received AZ in combination. 

**Table 5:** Multiplex bacterial and viral PCR results in the probable/confirmed COVID-19 patients.

	Confirmed	Radiologically diagnosed	Clinically suspected
Haemophilus influenzae	9*	5*	1
Streptococcus pneumoniae	4*	1*	0
Myvoplasma pneumoniae	0	1	0
Adenovirus	1	1*	0
Influenza B	1	0	0
Parainfluenza	1	0	0
Coronavirus	0	1	0
Total cases	15	7	1

* Two patients had nasopharyngeal swab PCR positive for H. influenzae, and S. pneumoniae, and one positive for H. influenza and adenovirus simultaneously.

The median duration of hospitalization was 4 days (0–28) (Table 6). A total of 15 patients (8.5%) were transferred to the ICU because of worsening respiratory function. Of the patients, 165 (93.7%) were discharged from the hospital, 4 (2.2%) died outside the ICU, and 5 were still in the ICU at the time of writing. Among deceased patients, 3 were confirmed cases, and one was radiologically diagnosed. Patients who were classified as having severe pneumonia had a higher duration of hospitalization, higher rate of ICU transfer, and higher rate of mortality. 

**Table 6:** The characteristics of the hospitalization period of probable/confirmed COVID-19 patients.

	Totaln: 174	Mild diseasen: 35	Moderate diseasen: 107	Severe diseasen: 32	P-value
Duration of hospitalization, days*	4(0–28)	3.5(0–12)	4(1–15)	7.5(2–28)	<0.001
Transferred to ICU, n (%)	15 (8.5)	2	5	8	0.001
Duration from hospitalization toICU transfer, Days*	5 (0–9)	6 (0–6)	4 (0–7)	5(0–9)	0.139
Discharge, n (%)	165 (93.7)	34	105	26	0.001
Ongoing hospitalization, n (%)	5 (2.8)	1	1	3	
Exitus, n (%)	4 (2.2)	0	0	4	

*Median (minimum–maximum)

Among the 4 deceased patients, one patient was 59 years old with Child class C liver cirrhosis, one was 74 years old with infective endocarditis and septic embolization, one was 83 years old with chronic renal failure, and the last patient was 92 years old with aplastic anemia. 

A total of 166 patients (95.4%) received HQ alone or in combination: 23 (13.2%) patients received HQ alone, 113 (64.9%) received HQ plus AZ, and 30 received HQ plus AZ plus FAV (Table 7). FAV was used in a total of 32 (18.4%) cases. Two patients received FAV monotherapy, whereas the remaining 30 received FAV as a sequential (n = 21) to the initial regimen or in combination (n = 9). Lopinavir/ritonavir was used in 3/174 in patients. One was a woman with an 18-week pregnancy and others received LPV/r after suboptimal response to initial HQ + AZ as recommended by the national guidelines. Patients received prophylactic anticoagulation with low molecular weight heparin according to national guidelines recommendation.

**Table 7:** The comparison of outcomes with different therapeutic regimens.

	HQ	HQ plus AZ	FAV-containing regimen	P-value
Number of cases, %	23 (13.2)	113 (64.9)	32 (18.4)	
Diagnostic criteriaConfirmed casesRadiologically diagnosedClinically suspected	1634	74318	2750	<0.001
Diseases severity, nMild ModerateSevere	1553	158612	31514	<0.001
Nausea/vomitingElevation of transaminases	11	53	510	0.038<0.001
Median time to defervescence, days*	1 (0–4)	1 (0–11)	3 (0–8)	<0.001
Median time to clinical improvement on therapy*, days	1 (1–6)	1.5 (1–11)	6 (1–20)	<0.001
Median duration of LOS, days	2 (1–21)	4 (1–15)	7.5 (2–24)	0.001

* Treatment response analysis was made in 165 patients who had been discharged.

Nausea/vomiting were a problem in 11/162 (6.3%) patients. Of 165 patients who had a control transaminase level, 16 (9.2%) had elevated transaminases which tended to normalize in the follow-up. In the patients who were in the FAV-treated group, both adverse reactions were more frequent: nausea/vomiting in the HQ group and HQ plus AZ group were 1/23 (4.3%), 5/106 (4.5%), whereas 5/28 (17.9%) in the FAV-containing regimen (P: 0.038). Transaminase elevation in the HQ group and HQ plus AZ group were 1/22 (4.5%), 3/105 (2.9%), whereas it was present in 10/28 (35.7%) in the FAV-containing regimen (P < 0.001). There was no significant QT prolongation, or arrhythmia in this case series. None of the patients discontinued antiviral therapy due to an adverse reaction. 

Four patients (2.2%) died. Five were still in the ICU at the time of writing. The median time to clinical improvement on therapy was 2 (1–20) days, and to defervescence was 2 (2–12) days (Table 7).

The median duration of hospitalization was different in three treatment groups (P: 0.001). The HQ group had the minimum, and the FAV group had the maximum duration of hospital stay (2 days vs 7.5 days, P < 0.001). There was also a significant difference between the HQ and HQ plus AZ group in terms of duration of hospitalization (P < 0.001).

## 4. Discussion

In this case series with prospective data collection, we summarized the characteristics, treatment regimens, and outcomes of the 174 probable/confirmed COVID-19 patients admitted to a Turkish university hospital consecutively during the pandemic. Among the study group, 69% were confirmed, and 31% were probable cases. ICU transfer rate was 8.5%, and the overall case fatality rate was 2.2%. 

The median age of the patients was 45.5 years, and only 13.8% of the patients were older than 65 years of age. The relatively younger age profile of our cohort may possibly be explained by the fact that Turkey has one of the youngest populations among OECD countries OECD Data [online]. Website: https://data.oecd.org/pop/young-population.htm#indicator-chart [accessed 06 June 2020].. In addition, early nationwide strict lockdown procedures were applied for those >65 years old possibly leading to a decreased exposure rate in this age group. On the other hand, the patients with severe pneumonia at hospital admission were older than those with mild or moderate disease (median age 56.5, vs 44, and 42 years old, respectively, P: 0.003).

Exposure through international travel was noted in only one case in this study. This could be related to the international travel ban to the epidemic regions in the world issued on February 2020, and suspension of all domestic and international flights after the identification of the first case on March 10, 2020. More than half of the patients (52.9%) had no known contact with a confirmed COVID-19 case. This highlights the current challenges of prevention of viral transmission in the population. 

Hypertension (18.9%) and diabetes mellitus (14.9%) were the two leading comorbidities. However, these rates are within the limits of the estimated prevalence in the whole population for both diseases World Health Organization (2020).COVID-19: Surveilllance, Case Investigation and Epidemiological Protocols [online]. Website: https://www.who.int/emergencies/diseases/novel-coronavirus-2019/technical-guidance-publications?publicationtypes=df113943-c6f4-42a5-914f-0a0736769008 [accessed 06 June 2020]., Arıcı M, Altun B, Erdem Y, Derici Ü, Nergizoğlu G et al. Prevalence, awareness and treatment of hypertension in Turkey (2002) [online]. Website: http://www.turkhipertansiyon.org/pdf/Turk_Hipertansiyon_Prevalans_Calismasi_Ozeti-1.pdf [accessed 06 June 2020] [4]. One-third (5/15) of those who were transferred to the ICU had hypertension.

Our study design does not enable us to determine the poor prognostic factors in the course of COVID-19 infection, but the higher frequency of diabetes mellitus or hypertension among patients who needed to be transferred to the ICU supports the previous observations [5–7]. A malignant disease was present in 6% of 5700 COVID-19 patients in New York City [8]. Malignancy was present in six (3.4%) of our patients, 4 had severe pneumonia, and unfortunately 2 died. 

In a recent paper, Farsalinos et al. pointed out the lower rates of smokers among COVID-19 patients [9]. In our study, smoking was observed in 32.2% of the patients, higher than previous studies [8–11]. According to the report made by the Turkish National Statistical Institute, the rate of smokers in the adult population was 23.8% in 2012 Global Adult Tobacco Report (2012) (in Turkish) [online]. Website: https://www.tuik.gov.tr/PreTablo.do?alt_id=1042 [accessed 06 June 2020]. . We found that 7 of 15 patients transferred to the ICU were active smokers. The relationship between smoking and the severity of COVID-19 should be clearly understood in further and larger case series or case-control studies.

In this study, the median duration of hospital stay was 4 days, comparable to the recently reported New York City (NYC) cohort of 5700 patients (4.1 days) [8]. Similarly, 13.2% were transferred to ICU (14.2% in NYC cohort) and case fatality rate was 2.2% (4.8% in NYC cohort). Not surprisingly, patients with severe pneumonia at the time of admission had a higher rate of ICU transfer, required ventilation support, and higher case fatality rate. Mortality in COVID-19 patients has been reported between 1.4% and 15% in different case series Centers for Diseases Control and Prevention (2020). Coronavirus Disease 2019 (COVID-19) [online]. Website https://www.cdc.gov/coronavirus/2019-ncov/covid-data/covidview/ [accessed17 May 2020]. [8,11–13]. We must underline that in those case series, the majority of the patients were still hospitalized at the date of closure of the database. The overall mortality rate is 2.7% in Turkey11 Republic of Turkey Ministry of Health (2020). Turkey COVID-19 patient table, 06 June 2020, [online] . Website: https://covid19.saglik.gov.tr [accessed 06 June 2020]..

Another respiratory bacterial and viral pathogen was detected by multiplex PCR in 19 and 4 patients, respectively. An early report showed that coinfection with another respiratory pathogen was frequent (14/32) in confirmed COVID-19 patients [14]. In subsequent series, coinfection rates were reported between 2 % and 8%, whereas Zhu et al. reported rates of coinfection as high as 94.2% in COVID-19 patients, leading to the recommendation of empiric antiinfluenza and antibacterial treatments [15]. In our study, coinfection with a respiratory pathogen was detected in 23 patients (13.2%). Low coinfection rates in our patient population discourage use of empiric antimicrobial treatment in COVID-19 patients and favors current recommendations according to the Turkish National Guidelines. 

Cytokine storm is one of the main drivers of COVID-19–related mortality [16]. It can be predicted by some laboratory values including lymphocyte count, CRP, D-dimer, and ferritin levels. Similar to previous reports [5–8,17–19], we found lower lymphocyte, high leukocyte and neutrophil counts, higher NLR, higher serum CRP, procalcitonin, d-dimer, ferritin, LDH, troponin levels were associated with severe COVID-19 disease. Unfortunately, we could not measure cytokine levels in this study, a limitation for our results. In addition, we could not draw a definitive conclusion on the effectiveness of prognostic markers because the rate of ICU transfer, critical patients, and mortality were low in this study. 

Despite scarcity of convincing and evidence-based data, our COVID-19 treatment strategy followed the updated guidelines of the Turkish Ministry of Health and in-hospital guidelines developed by a multidisciplinary team. Patients with pneumonia received HQ and AZ in combination. Favipiravir was not available in large quantities, and restricted to use only in critically ill patients who required ICU in the early days of the pandemic. After April 14, 2020, National Guidelines amended recommendations to use FAV in patients with bilateral pneumonia. In our case series, 13.2% patients received HQ alone, 64.9% HQ plus AZ, and 18.4% were treated with regimens including FAV without any significant adverse effects during the hospitalization period. The durations of hospital stay, times to defervesce, and symptom improvement were longer in the FAV-receiving group but similar between HQ, and HQ plus AZ group. This outcome is not surprising because FAV was prescribed to patients failing under first-line regimen (HQ and/or AZ) or patients who deteriorated during follow-up. Although HQ monotherapy was reserved for patients without pneumonia and mild symptoms, we could not detect any difference between HQ and HQ plus AZ groups in terms of symptom resolution. We must emphasize that this is an observational descriptive study, not designed to compare treatment regimens, so these results should be interpreted cautiously. There is no efficient or gold-standard treatment for COVID-19 at the moment Bhimraj A, Morgan RL, Shumaker AH, Lavergne V, Baden L et al. Infectious Diseases Society of America Guidelines on the Treatment and Management of Patients with COVID-19 [online]. April 21, 2020. Website www.idsociety.org/COVID19guidelines [accessed 06 June 2020]. . A large observational study from France demonstrated favorable results with HQ treatment; virological cure was obtained in 91.7% of patients within 10 days whereas the mortality was 0.75% [20]. However, this was a retrospective study with no randomization. Several other trials failed to show any benefit from HQ treatment in COVID-19 patients [21–23]. Data regarding FAV is still scarce and confined to low-quality studies [23–25]. The results of the ongoing randomized controlled clinical trials are expected to clarify the confusion related to treatment of COVID-19. 

Our study has several limitations. Firstly, it is a single-center observational study with a relatively low number of patients. Patients were not randomized for treatment, but were categorized according to severity when allocated to different therapeutic regimens. Thus, a true comparison between different regimens was not possible. We could not perform a risk factor analysis for disease progression, or outcomes as the number of patients who had a complicated clinical course was low.

In conclusion, we observed a low mortality rate in a series of 174 patients with COVID-19 admitted early to the hospital and given antiviral therapy. Our results may warrant further investigation of the combined effects of these practices. 

## Informed consent

The institutional review board was informed (Hacettepe Üniversitesi Girişimsel Olmayan Araştırmalar Etik Kurulu, GO 20/354) and informed consents were obtained from the participants.
